# A Novel Self-Assembling DNA Nano Chip for Rapid Detection of Human Papillomavirus Genes

**DOI:** 10.1371/journal.pone.0162975

**Published:** 2016-10-05

**Authors:** Xin Li, Yanbo Li, Li Hong

**Affiliations:** Department of Gynecology 2, Renmin Hospital of Wuhan University, Wuhan 430060, Hubei, China; "INSERM", FRANCE

## Abstract

Rapid detection of tumor-associated DNA such as Human Papillomavirus (HPV) has important clinical value for the early screening of tumors. By attaching oligonucleotides or cDNA onto the chip surface, DNA chip technology provides a rapid method to analyze gene expression. However, challenges remain regarding increasing probe density and improving detection time. To address these challenges, we proposed a DNA chip that was self-assembled from single stranded DNA in combination with high probe density and a rapid detection method. Over 200 probes could be attached to the surface of this 100-nm diameter DNA chip. For detection, the chips were adsorbed onto a mica surface and then incubated for ten minutes with HPV-DNA; the results were directly observable using atomic force microscopy (AFM). This bottom-up fabricated DNA nano chip combined with high probe density and direct AFM detection at the single molecule level will likely have numerous potential clinical applications for gene screening and the early diagnosis of cancer.

## Introduction

Assessing the presence and function of high-risk human DNA such as papillomavirus (HR-HPV) DNA is important for effective cancer screening. The specific detection of HPV genotypes 6/11 and/or 16/18 in clinical samples is of crucial importance. It is reported that in 65% of women with atypical cytology and of the HPV positive cases, 68% were HPV 16/18, 22% HPV 6/11 and 10% of mixed types. The detection and typing of HPV-DNA in cell scrapings is a relatively fast and non-invasive procedure which complements cytology, colposcopy and histology and should be useful in further studies of the natural history of different HPV infections and their role in cervical cancer [1–4]. HPV DNA detection and genotyping is useful in the early diagnosis of cancers such as cervical, tonsillar, and head and neck cancers. Previously, DNA chips based on microarrays and fluorescently labeled probes have been employed in the detection of HPV DNA [3,4]. However, for screening large numbers of samples, an improvement of detection efficiency is required. One promising strategy to address this issue involves the use of newly developed DNA nanotechnology, which provides a novel bottom-up methodology for the fabrication and detection of target DNA at the single molecule level.

The manufacture of functional devices on a nano scale represents a significant but challenging target for the field of nanotechnology. Programmable DNA self-assembly has been demonstrated by several studies [5–10] to be a promising method for achieving this goal. For example, DNA tile self-assembly uses the hybridization of several short strands to fold a rigid structure, termed programmable DNA tiles [11], which can be used to assemble 2D and 3D patterns. A related technique is DNA origami [12], which uses hundreds of short staple strands to fold a long scaffold strand into any desired shape.

Nanoscale DNA structures such as those generated by folding can be functionalized by certain biological molecules and quantum dots including DNA [13], RNA [14], proteins [15–17], and gold nanoparticles [18,19], respectively, which can be attached to the specific DNA strands. For example, Ke et al. [20] developed a method to detect target DNA with high accuracy using probe tiles exhibiting efficient self-assembly. In this method, the consideration of steric hindrance between the DNA origami and the targets resulted in a more advanced detection system. However, owing to the symmetry of the folded DNA substrate, a coordinate index point was essentially pre-assembled on the origami surface. To address this issue, an asymmetric origami construct was manufactured to eliminate the index point [21]. Associating the thought with strand displacement, Zhang et al. [22] developed a novel scheme in which target DNA strands replaced the biotinylated reporter strand, thus causing the light dots that appeared on the origami surfaces under observation using atomic force microscopy (AFM) to disappear when target strands were present. Further research demonstrated a linear relationship between the target concentration when RNA was utilized and the percentage of light dots displaced, which implied a potential quantitative measurement of target RNA *in vivo* [23]. The development of a DNA origami binding assay for the capture and detection of thrombin, a kind of protein, in cell lysates [24] represented another innovative application of this technology.

Compared with conventional DNA or micro-RNA detection methods such as northern blotting [25], real-time polymerase chain reaction (PCR) [26,27], and the high-throughput microarray approach [28], the DNA origami-based detection strategy has several advantages such as low cost, relatively accurate precision, and a low requirement for sample purity [23]. However, the general application of this method would require increased hybridization efficiency [23] and improved signal readout methodology [22]. Relevant questions include whether it would be possible to improve the efficiency of single molecule detection using AFM and how the detection result could be stabilized. Furthermore, a relevant goal would be to develop a universal method capable of detecting any specific sequence of clinical interest. To address these challenges, we designed a DNA chip based on DNA origami that was able to directly detect HPV DNA, an important target for gynecologic diagnoses, with rapid determination of the results by AFM over several minutes.

## Materials and Methods

### Ethics Statement

The study was approved by the insititutional review board of Renmin Hospital of Wuhan University, Wuhan, China. All patients provided written informed consent.

### Preparation of Human clinical samples

In order to detect human clinical samples, two clinical samples in our hospital were selected (refer to supplement information S1 Fig). Both these two samples were detected by Luminex® 200™ with TellGen HPV DNA test Kit bought from TellGen Corp. (Shanghai, China), one selected sample is HPV 16 positive as the DNA target sample for our nanoscale DNA chip, while another is HPV 16 negative as a control sample. The samples for our nanoscale DNA chip detection is prepared as follows:

1. Put 400 μL collected crevical cells samples into test tube, centrifuge at 13000 rpm for 5 minutes then discard supernatant.
2. Add 200 μL Tellgen nucleic acid releasing agent to the samples, after mixture, samples were incubated at 95°C for 15 minutes
3. Centrifuge at 13000 rpm for 5 minutes, decant the supernatant and discard the pellet, kept samples at -20°C for further steps.
4. PCR with TellGen HPV DNA test Kit. Mixture 5 μL DNA sample with 10 μL PCR pre-mixture and 0.8 μL Taq in TellGen HPV DNA test Kit. The mixture samples were put in Biorad T100 PCR instrument and PCR for about one hour as programs in supporting information S1 Table.
5. The PCR results were diluted 10 times to 150 μL with TAE buffer and kept at -20°C for further nanoscale DNA chip detection without purification.

### Fabrication of a DNA chip for HPV detection

The nanoscale DNA chip developed in this study was based on a rectangle DNA folded origami structure with 216 staples. The software CaDNAno [4] was employed to aid with the visual design of the 2D DNA origami. We fabricated a nanoscale platform with a relatively rigid rectangle structure generated by the folding of a 7250-nt-long M13mp18 with the staple strands.

An HPV probe (‘5′-GTCATTATGTGCTGCCATATCTACTTCAGA-3′’) was attached to the DNA chip by extending two kinds of staples that were located in columns 12 and 14; each kind of staple included 12 staples. The HPV probe was divided into two equal parts. The first half of the probe sequence was added to the 3’ end of the staples in column 12 and the second half of the probe sequence was added to the head of the 5’ end of the staples in column 14. The distance between the two neighboring first-half and second-half probes was 32 nt. Two Ts were inserted into all the probe staples between the staple sequence and the probe sequence to provide enough flexibility to increase the hybridization efficiency of the probe strands and the target strands. The final DNA chip incorporating the probes to detect the target HPV strand was prepared by replacing the staple strands in column 12 and 14 with the extended probe staple strands.

All staple strands were purchased from Shanghai Sangon Biotech Company (Shanghai, China) with high affinity purification. The scaffold strand M13mp18 was purchased from New England Biolabs Inc. (Ipswich, MA, USA).

The DNA chip samples were prepared by mixing the scaffold M13mp18 strand and 180 normal staple strands and 24 extended probe staples in TAE buffer (12.5 mM Mg^2+^, 20 mM Tris pH 8.0, and 2 mM EDTA). The final concentration of the scaffold strand was 10 nM and the ratio of scaffold strands to all staples was 1:10. The mixture was annealed in a PCR instrument from 94°C to 16°C at a cycling speed of −1°C per minute, and finally stored at 4°C.

The sample purity is of crucial important to the success detection of target DNA with our DNA chip detection method. We employed two methods to purify our DNA chip samples before being observed using AFM, which can prevent the surplus staple strands with extended probe sequence from interference the experiment result by interact with target strands. The first method is to lower the concentration ration between staple strand and scaffold strand from 100:1 in previous works [12, 23] to our 10:1. Although, the low concentration of staple strands will surely lower the production of DNA origami, we amend this disadvantage by anneal with longer time and purify the staples before anneal. The second method is to use Microcon YM-100 100kDa to purify all samples and get rid of the rest short staple strands (generally less than 30kDa) before incubation.

### AFM observation and detection

To observe the sample of nanoscale DNA chips by AFM, 2 μL DNA chips (10 nM) incorporating the HPV probes was dropped onto a fresh cleaved mica surface. The sample was left at room temperature for 2 minutes for absorption, then 30 μL TAE buffer was added to the sample and the AFM tip, respectively. AFM results were obtained using a Bruker Multimode-8 Nanoscope (Billerica, MA, USA) under ScanAsyst mod with a ScanAsyst Fluid+ tip. The engaged force of the ScanAsyst mod was maintained under 0.02 V and the scan rate under 1 HZ during scanning.

The mica incubation strategy was employed to detect target the DNA. After withdrawing the AFM tip, the buffer on the mica scaffold was absorbed with filter paper and 30 μL target DNA in TAE buffer (100 nM) was dropped onto the mica. The sample and target DNA were incubated at room temperature for 5 minutes. After the on-mica incubation, the sample of the DNA chip with the target DNA was scanned using AFM.

## Results and Discussion

In this study, we fabricated a nanoscale platform based on a rectangle DNA folded origami structure with 216 staples that could detect and analyze disease related genes and polypeptides.

**Fig 1:**
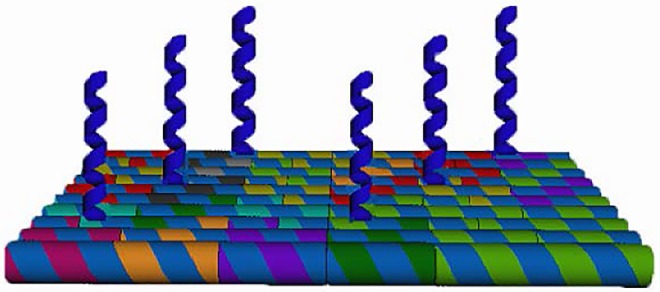
Scheme of the DNA chip and probes.

In principle, DNA probes can be located in arbitrary positions on folded DNA structures by the elongation of specific staple strands that fold and anchor the DNA origami. These DNA probes can be designed as complementary sequences of target DNA to detect specific gene expression. In addition, these projecting single-stranded DNAs can also be functionalized with fluorophores, nanoparticles, or protein polypeptides that allows the DNA origami to serve as a nanoscale lab on a chip. The scheme of the DNA chip and attached DNA probes is shown in Fig 1.

In this study, we use the probes on our DNA chip to detect HPV DNA, which is positively associated with cervical cancer and thus is of relevance in gynecological clinical diagnosis. However, two primary challenges needed to be addressed regarding the detection of HPV DNA with our nano DNA chip. Firstly, for single strand DNA probes on a DNA origami platform, the length of the DNA probe is restricted by the length of the staple strands. On the other hand, for a target DNA such as HPV, the specific gene sequence needing to be screened for effective clinical detection is relatively long. Typically, the length of a staple strand is 32 nt long whereas the extended staple strand including probe sequence should not exceed 60 nt to minimize the costs of synthesis and retain stability. As the probe sequence for HPV DNA was 30 nt long, the maximum length of probe portion of the staple strand was not sufficient for HPV detection. Secondly, after hybridization, the long double stranded DNA (dsDNA) consisting of target and probe DNA had only one side attached to the DNA chip. This final dsDNA helix was therefore unstable under AFM during scanning because the unattached side of the final double stranded DNA would swing back and forth. This effect of the dsDNA helix was driven by the AFM tip during the forward and revised procedure and resulted in blurred and indeterminate data, which is not appropriate for clinic diagnosis.

**Fig 2:**
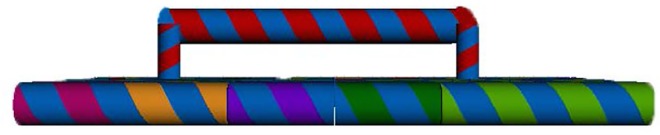
Scheme of separated DNA probes for a single long target sequence. This strategy resulted in a firmly anchored helix on the DNA chips that is appropriate for AFM detection.

We proposed a single solution to solve these two problems as follows. The method was to divide the 30 nt HPV DNA into two or more sections and to then locate these neighboring parts on the continuous staples on a DNA chip. With this approach we were able to detect target DNA sequences of any length within the length restriction of the DNA staples. Furthermore, the final dsDNA was attached to the chip at 2 or more positions, which firmly anchored the resulting double helix onto the DNA chip and resulted in stable AFM scan data. The scheme of this solution is shown as Fig 2.

**Fig 3:**
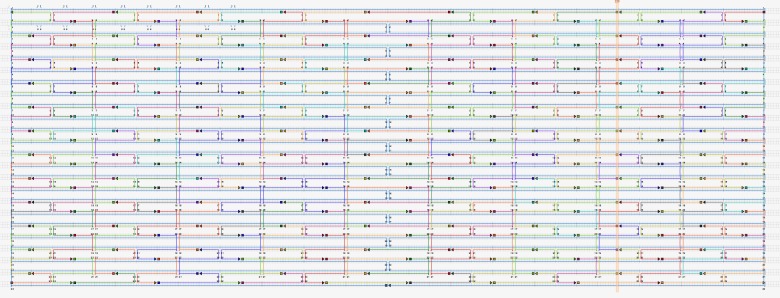
DNA chip structure and sequence of all staple strands.

To build the DNA nano chips, CaDNAno software, which can help to rapidly and accurately determine staple sequences according to the known scaffold sequence, was employed to aid with the visual design of the 2D DNA origami [4]. The final designed DNA chip structure consisted of 216 staples in 12 rows and 18 columns as shown in Fig 3. The DNA chip shown in Fig 3 is very much resemble the DNA origami previously described in ref [12]. It needs to clarify that our strategy with using extended staple strands of DNA origami in ref 12 as probes which can detect HPV DNA and other arbitrary target DNA strands as well. With this strategy, we transfer the the formal structural of DNA origami into our functional nano-device: DNA chip.

**Fig 4:**
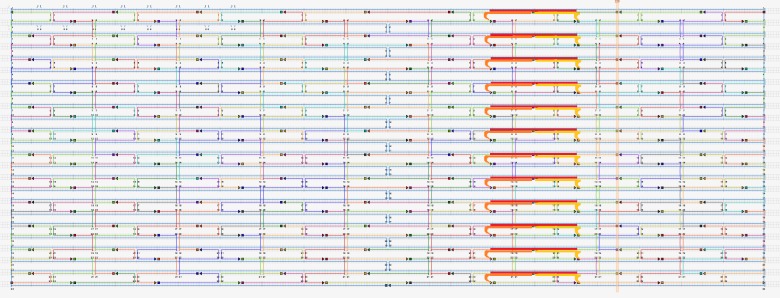
Scheme of a DNA chip with attached probes incubated with target DNA strands. The structure and sequence of all staple strands is shown.

**Fig 5:**
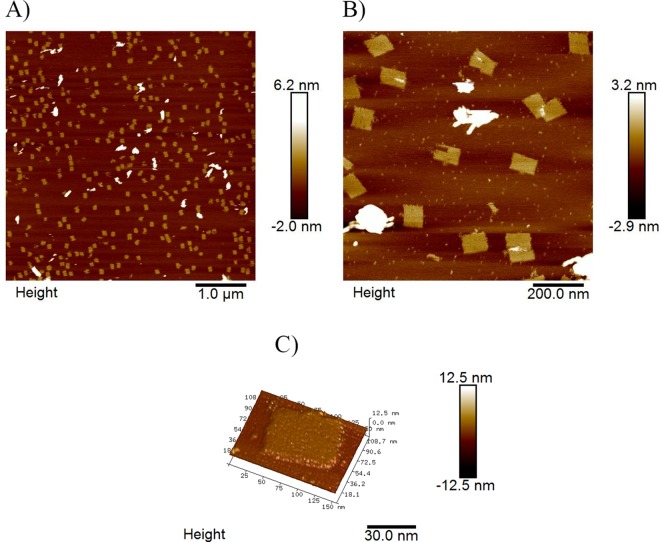
AFM height results of DNA chips in TAE buffer. A) Area scan (5 μm × 5 μm) of DNA chips. B) Zoomed in image (1 μm × 1 μm) of DNA chips. C) 3D AFM image of prepared DNA chips without target.

The design and position of the separated probes and the final double stranded structure after incubation is shown in Fig 4. AFM images of the generated DNA nano chips are shown in Fig 5.

The AFM results shown in Fig 4. of the DNA nano chips prior to target hybridization demonstrate that rectangular DNA chips could observed at the different scan resolutions. Approximately 20 DNA chips were present on a 1 μm × 1 μm area. The side length of one DNA chip was about 100 nm. Most DNA chips appeared well-formed and uniform, indicating that the extended probe strands of the selected staples did not seem to affect the assembly process of the DNA chips.

The surface of the well-formed DNA chips was flat. The probe strand was not detected under AFM, which might be because the single-stranded DNA does not have sufficient rigidity. In other words, the 15 nt long single stranded probes extending from the staples were not able to provide enough response to the AFM tip under the ScanAsyst mod to facilitate detection. Thus, the assembled DNA chip performed similar to previously described DNA origami constructs [12].

**Fig 6:**
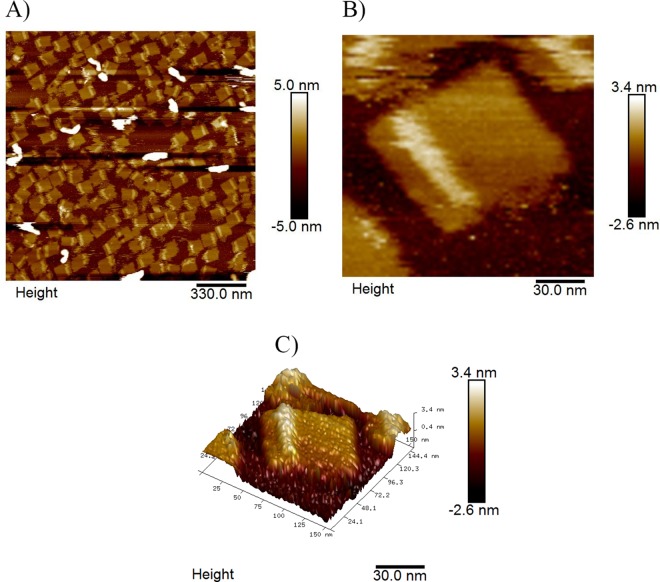
AFM result of DNA chips incubated with the target DNA. A) Area scan (2 μm × 2 μm) of DNA chips. B) Zoomed in image (150 nm × 150 nm) of DNA chips. C) 3D AFM image of DNA chips incubated with the target DNA.

Fig 6 shows the AFM results following target strand incubation. The expected dsDNA is observed on one side of the DNA chip. As illustrated in Fig 4, a total of 12 double strands are present on one side of the DNA chip with the same sequence and structure. The length of each dsDNA helix formed by two separated probes and one target strand is 32 bp. All 12 dsDNA helixes together form an elevated band on the top of the DNA chip with a width of about 11 nm and length about 60 nm, which is consistent with our AFM results as shown in Fig 6B and 6C.

The key point to get high resolution AFM results is the purification of the DNA chip samples. With the two purification methods previously described, we got high yield DNA chip samples and good detection result of target DNA with this DNA chip, as shown in Fig 6C, the height change of incubated probes with target DNA on DNA chip can be clearly detected by AFM with good resolution quality. Moreover, the AFM image quality in Fig 5 of DNA chips before incubation with target DNA also confirmed the high yield of our productions.

The high resolution AFM image shown in Fig 6C demonstrated that the detection of target HPV DNA sequence on our nanoscale DNA chip could be directly obtained by AFM tip scanning, which is very different from the technique used for previous DNA chips that utilized the fluorescently labeled DNA detection method. By attaching DNA probes onto a folded rectangle of DNA as the foundation for the DNA chip, a method for single molecule level incubation and detection of the target DNA is achieved. In comparison, hybridization at a single molecule level is difficult to detect by the fluorescence labeling method due to the resolution limitation of fluorescent microscope. Using the AFM height data, the single molecule incubation results could be easily detected from the rigid double strand structure that formed on the chip. Single stranded DNA and dsDNA on DNA chips after incubation exhibited significant differences in the height images obtained under AFM as shown by our results in Fig 5 and Fig 6, respectively.

Compared to previously utilized indirect DNA detection methods wherein target DNA eliminated the strand displacement that highlighted spots on DNA origami structures [22], our approach is direct and more efficient. The stability of the resulting dsDNA helix on the nano DNA chip is the key factor for the reliable direct detection of target DNA by AFM. As opposed to the traditional one-sided probe attachment method, which results in an unstable and blurred AFM scan image, we proposed a probe separation strategy. With this strategy, the resulting dsDNA helix consisted of two separated probes and one target strand that had two ends attached to the DNA chips via two kinds of staple probes. This dsDNA helix was stable on the surface of the DNA chips and able to withstand the scanning process of AFM.

Finally, the DNA chip we proposed here was self-assembled from single stranded DNA using a bottom-up method, which is very different from traditional DNA chips that are produced with top-down nanofabrication technology. With the bottom-up fabrication strategy, numerous nanoscale DNA chips can be self-assembled with programmable probes. In addition, the density of these probes can be far beyond that achieved by traditional top-down nanofabrication approaches, and the positions of the probes on the nanoscale DNA chips can be controlled in the range of nanometer precision. Furthermore, single molecule target genes can be directly detected by AFM without any additional DNA modifications, which might minimize the consumption of target samples as well as the detection time.

## Conclusion

In this study, we developed a nanoscale DNA chip that was able to efficiently detect HPV DNA. This DNA chip was directly self-assembled from single stranded DNA. By incorporating a bottom-up self-assembly strategy, the nano DNA chip was able to perform single molecule level detection of target genes with precision control of probe position in the nanometer range, which resulted in a very high density of probes on the mica scaffold surface. Furthermore, the detection results could be directly obtained by AFM after 5 minutes following mica incubation without any additional DNA modification such as the standard fluorescence labeling utilized for most traditional DNA chips, which is convenient for clinical gene screening applications.

With the foundation of a self-assembled DNA chip substrate and functional staples with single stranded probes, modifications such as attached polypeptides and other nano-particles might be fixed onto the DNA chip surfaces with nanometer precision control of their position in a programmable way. This DNA nanotechnology might also be potentially applied to research related to gene detection, gene sequencing, genetic screening, protein functionality, and nanometer circuits.

## Supporting Information

S1 Fig Human HPV Clinical Data. (TIF)

S1 File Supporting Information. (DOCX)

S1 Table PCR programs. (DOCX)
