# Supplementary figures and images for: A Novel Self-Assembling DNA Nano Chip for Rapid Detection of Human Papillomavirus Genes
Source: PLoS One. 2016 Oct 5;11(10):e0162975. doi: 10.1371/journal.pone.0162975 (PMC5051682; doi:10.1371/journal.pone.0162975)

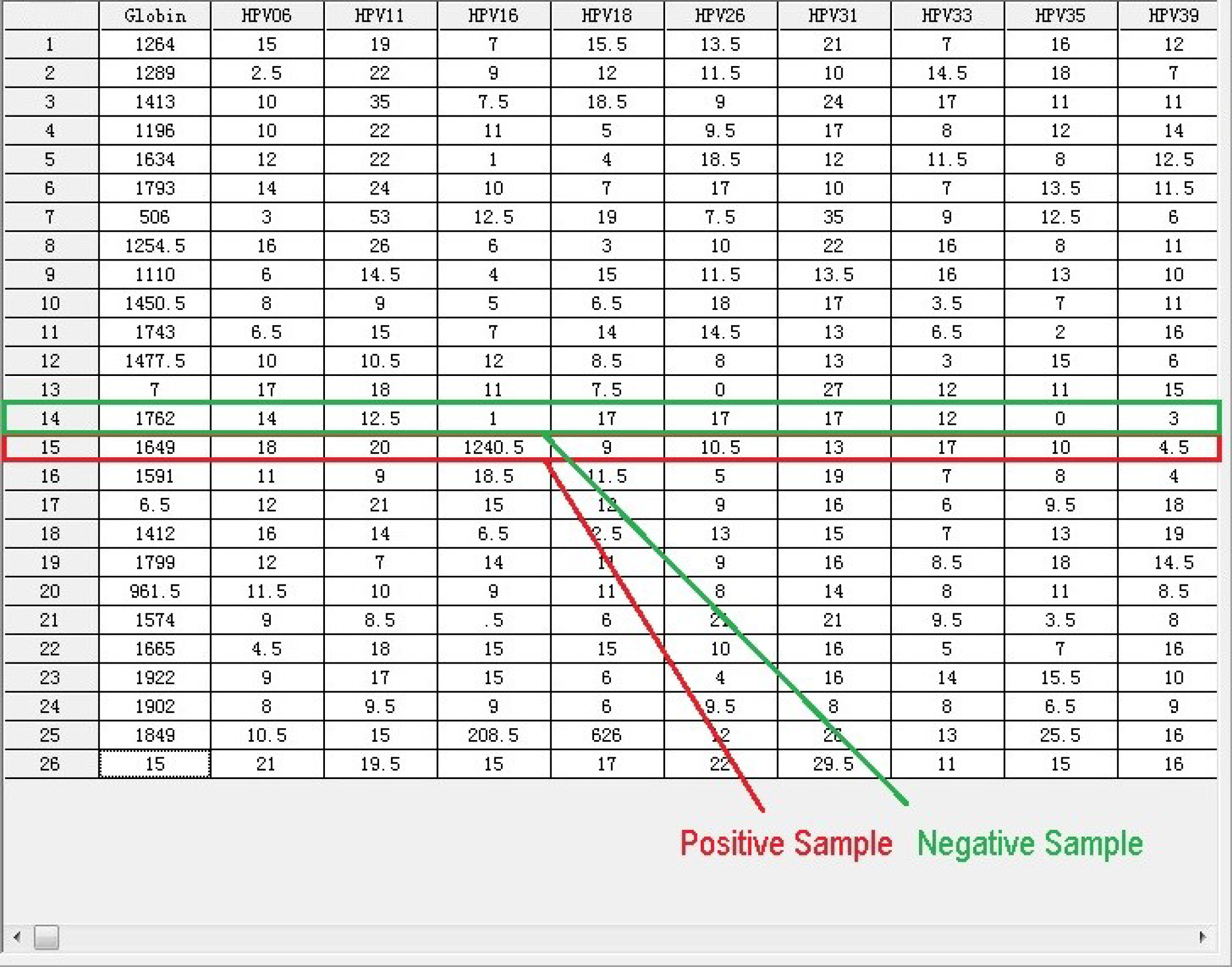

Supplement: S1 Fig — (TIF) [file pone.0162975.s001.tif]
