# Supplementary material for: A Novel Self-Assembling DNA Nano Chip for Rapid Detection of Human Papillomavirus Genes
Source: PLoS One. 2016 Oct 5;11(10):e0162975. doi: 10.1371/journal.pone.0162975 (PMC5051682; doi:10.1371/journal.pone.0162975)
Supplement: S1 File — (DOCX) [file pone.0162975.s002.docx]

**Supporting Information**

**Sample purification**

Our samples are purified with Microcon 100kDa YM-100 before incubation to get the best AFM results. 50μL sample was dropped into YM-100, the YM-100 was put into Eppendorf centrifuge 5415D and spin at 4,000 rpm for 1 hour. After that, 50μL TAE buffer was added to the sample and spin in Eppendorf centrifuge at 4,000 rpm for 1 hour. Finally, the liquid in the bottom of YM-100 was discarded, 50μL TAE buffer was added to the filtered sample shock for 10 seconds, then put the filter upside down and spin again at 4,000 rpm for 10 minutes. After filtered by 100kDa YM-100, most extra DNA strands less than 100k Dalton (Typically here less than 60 bp long single stranded DNA, which will be around 18k Dalton) were purified from the final sample, which will led to a clear and high resolution AFM image.

**Directly observe the incubation results under AFM**

AFM results were obtained using a Bruker Multimode-8 Nanoscope (Billerica, MA, USA) under ScanAsyst mod with a ScanAsyst Fluid+ tip. The engaged force of the ScanAsyst mod was maintained under 0.02 V and the scan rate under 1 HZ during scanning. To get the best AFM image, try to lower the engaged force to 0.01 V as far as possible, since the DNA chip structure is soft, and the double stranded incubation results of DNA probes is event softer. So the low engaged force of AFM tip is the key point to get the detection results directly under AFM.
