# Supplementary material for: A Novel Self-Assembling DNA Nano Chip for Rapid Detection of Human Papillomavirus Genes
Source: PLoS One. 2016 Oct 5;11(10):e0162975. doi: 10.1371/journal.pone.0162975 (PMC5051682; doi:10.1371/journal.pone.0162975)
Supplement: S1 Table — (DOCX) [file pone.0162975.s003.docx]

**S1 Table. PCR programs**

| Program | Cycles | Temperature (℃) | Incubation Time (min:sec) |
| --- | --- | --- | --- |
| 1 | 1 | 95 | 5 min |
| 2 | 5 | 95 | 30 sec |
| 2 | 5 | 58 | 30 sec |
| 2 | 5 | 72 | 30 sec |
| 3 | 35 | 95 | 30 sec |
| 3 | 35 | 55 | 30 sec |
| 3 | 35 | 72 | 30 sec |
| 4 | 1 | 72 | 3 min |
